# T58. SARCASM COMPREHENSION AS A SOCIAL COGNITION MEASURE IN SCHIZOPHRENIA – A SYSTEMATIC LITERATURE SEARCH AND META-ANALYSIS ON THE USE OF THE TASIT

**DOI:** 10.1093/schbul/sby016.334

**Published:** 2018-04-01

**Authors:** Alexander Rapp, Franziska Purr, Anne Felsenheimer

**Affiliations:** University of Tuebingen

## Abstract

**Background:**

Social cognition tasks with higher ecologically validity could be helpful both as an outcome measure for training and for social cognition impairment in schizophrenia. The comprehension of sarcasm and irony is a candidate for a valid, replicable task.

**Methods:**

Tests and paradigms as well as studies in schizophrenia are available in English, Dutch, German, Italian, Greek, Japanese and other languages. The Awareness of Social Inference Test (TASIT) (McDonald et al.,J head trauma rehabil 2003,) is currently the by far most applied paradigm. Here, we present a systematic literature research and meta-analysis on application of these paradigms in patients with schizophrenia.

**Results:**

25 studies with data from n=2185 patients with schizophrenia and n=1474 controls used the TASIT. This exceeds the numbers for other irony comprehension paradigms. Separate meta-analyses were calculated for the “sarcasm-enriched” and “sarcasm-minimal” subtests with data from 5 different English language studies. In both subtests, patients with schizophrenia showed significant impairment. Non-English translations of the TASIT show a comparable picture. Longitudinal data are available from 4 studies. Studies in high risk populations showed mixed results, however the TASIT is included in longitudinal cohort studies such as NAPLS-2.

**Discussion:**

We discuss differences with other task such as paradigms without prosodic or face information or the available fMRI investigations.

